# The Distribution of M2 Macrophage and Treg in Nasopharyngeal Carcinoma Tumor Tissue and the Correlation with TNM Status and Clinical Stage

**DOI:** 10.31557/APJCP.2021.22.11.3447

**Published:** 2021-11

**Authors:** Siti Hamidatul Aliyah, Yustina Nuke Ardiyan, Iffah Mardhiyah, Camelia Herdini, Ery Kus Dwianingsih, Sumartiningsih Aning, Niken Satuti Nur Handayani, Widya Asmara, Jajah Fachiroh, Dewi Kartikawati Paramita

**Affiliations:** 1 *Postgraduate Program, Faculty of Biology, Universitas Gadjah Mada, Yogyakarta, Indonesia. *; 2 *Pharmacy Program, Sekolah Tinggi Ilmu Kesehatan Harapan Ibu, Jambi, Indonesia.*; 3 *Department of Histology, Faculty of Medicine, Duta Wacana Christian University, Yogyakarta.*; 4 *Faculty of Dentistry, Universitas Gadjah Mada, Yogyakarta, Indonesia. *; 5 *Department of Otorhinolaryngology Head and Neck Surgery, Faculty of Medicine, Public Health and Nursing, Universitas Gadjah Mada, Yogyakarta, Indonesia. *; 6 *Department of Anatomical Pathology, Faculty of Medicine, Universitas Gadjah Mada, Yogyakarta, Indonesia. *; 7 *Integrated Research Laboratory, Faculty of Medicine, Public Health and Nursing, Universitas Gadjah Mada, Yogyakarta, Indonesia. *; 8 *Department of Microbiology, Faculty of Veterinary Medicine, Universitas Gadjah Mada, Yogyakarta, Indonesia. *; 9 *Department of Histology and Cell Biology, Faculty of Medicine, Public Health and Nursing, Universitas Gadjah Mada, Yogyakarta, Indonesia. *

**Keywords:** CD163, FoxP3, M2 Macrophage, Treg, Nasopharyngeal Carcinoma, Epstein, Barr virus

## Abstract

**Objective:**

This study aimed to identify the distribution of M2 macrophage and Treg in Nasopharyngeal Carcinoma (NPC) tumor tissue samples. The presence of these two groups of cells was further correlated to clinical stage, tumor size, the lymphatic node involvement, and metastasis.

**Methods:**

The total of 50 formalin-fixed paraffin-embedded (FFPE) NPC tissue samples was collected retrospectively (27 samples) and prospectively (23 samples). Samples were FFPE tissue slices. Immunohistochemistry was done on the FFPE tissue slides using anti-CD-163 and anti-FoxP-3 antibodies for M2 macrophage and Treg detection, respectively. The M2 macrophage interpretation was performed by eye-balling method and the score was divided into 0 (negative), 1 (scant), 2 (focal), and 3 (abundant). The average number of Treg FOXP3+ cells in 5 high power fields (HPF) was calculated. The relationship of M2 macrophage and Treg was tested with Spearman’s correlation. The relationship between M2 macrophage and Treg with clinical stage, tumor size, node involvement and metastasis was tested by chi square, with p<0.1.

**Results:**

M2 macrophage and Treg were positive correlated (r=0.469, p<0.001). The presence of M2 macrophage and regulatory T cell (Treg) was significantly correlated to tumor size (p= 0.091 for M2 macrophage and p=0.022 for Treg) and clinical stage (p= 0.030 for M2 macrophage and p= 0.002 for Treg), but did not correlate with lymphatic node involvement and metastasis.

**Conclusions:**

In Epstein-Barr virus related NPC tumor microenvironment, the presence of M2 macrophage was correlated with Treg, and both types of the cells were correlated with tumor size and clinical stages.

## Introduction

Nasopharyngeal carcinoma (NPC) is a type of head and neck cancer in nasopharyngeal epithelial tissue. The incidence of NPC worldwide is rare, but endemic in several regions, such as Southeast Asia, China, Northeast India, North Africa and in the Inuit population (Eskimos) in Canada and Alaska (Parkin et al., 2005). In Indonesia with a population of around 266 Million, NPC is estimated to be ranked as fifth of all malignancies, with an incidence rate of 6.6 per 100,000 population (Bray et al., 2018). The development of NPC in endemic areas is closely related to Epstein-Barr virus (EBV) infection and chronic inflammation. Tumor cells in NPC interact with surrounding cells to form a microenvironment that supports tumor development.

Epstein-Barr Virus encoded RNAs (EBERs) is the most commonly found viral transcript in cells of latently infected with EBV, including NPC cells, and the encoded gene is transcribed by RNA polymerase III (Takada, 2012). EBERs can induce expression of proinflammatory cytokines in NPC cells, which is dependent on retinoic acid-inducible gene I (RIG1) and toll-like receptor 3 (TLR3) activation (Duan et al., 2015; Li et al., 2015). Proinflammatory cytokines resulting from the interaction of EBERs and TLR-3 attract M2 macrophage that leads in Treg recruitment (Li et al., 2015). Proinflammatory cytokines can modulate polarization of macrophages primarily into M2 macrophage. The density of M2 macrophages in the tumor microenvironment has been found as a poor prognostic marker for various carcinomas (Cho et al., 2012). It is thought that M2 macrophages promote tumor growth by suppressing the immune system (Ooft et al., 2018; Wang et al., 2017). M2 macrophages have been shown to play a key role in immune suppression (Chen et al., 2012). M2 macrophage can recruit mature Treg by secreting cytokines such as CCL22 and CCL18, and M2 macrophages can modulate the conversion of naïve T cells into Treg by secreting transforming growth factor-beta (TGF-β) and Interluekin-10 (IL-10) (Mantovani et al., 2002). Tregs are directly involved in promoting angiogenesis in the tumor microenvironment, and also promote cancer through immune escape (Facciabene et al., 2012). Research on the relationship of M2 macrophage and Treg with prognosis and severity of several cancers has been widely studied. However, research of these two cells in the microenvironment of NPC is still limited; therefore, it needs to be studied further. This study aimed to identify the distribution of M2 macrophage and Treg in the cancerous tissue samples of NPC. The presence of these two groups of cells was further correlated to staging, tumor size, the lymphatic node involvement, and metastasis.

## Materials and Methods

This research was a descriptive observational study with a cross-sectional approach. Sampling technique using consecutive sampling with a minimum sample size for M2 Macrophage was 27 samples and Treg was 21 samples with the proportion of M2 macrophage (65%) or Treg (77%) in NPC (Ooft et al., 2017) . 

*Study subjects*

The total of 50 formalin-fixed paraffin embedded (FFPE) samples were collected retrospectively (27 samples) and prospectively (23 samples) from the Department of Pathological Anatomy, Dr. Sardjito General Hospital, Yogyakarta, Indonesia. All of the samples were derived from patients in Dr. Sardjito General Hospital, Yogyakarta, Indonesia, at their first visit prior to any therapeutic treatment. The retrospective samples were collected from 2008 – 2010 and the prospective samples were collected during April 2019 to February 2020. This study was approved by The Medical and Health Research Ethics Committee of the Faculty of Medicine, Public Health and Nursing, Universitas Gadjah Mada, Yogyakarta, Indonesia (No. KE/FK/0050/EC/2019).

*Immunohistochemistry (IHC) staining*

The FFPE samples were sliced at 3µm thick. Immunohistochemistry staining was performed by using Mouse and Rabbit Specific HRP/DAB IHC Detection Kit-Micro-polymer (Cat No: ab236466, Abcam, Cambridge, UK). Antigen retrieval was performed by using a Tris EDTA pH 9,0 for M2 macrophage and citrate buffer PH 6,0 for Treg. Monoclonal anti-CD163 antibody (clone EPR19518, Abcam, Cambridge, UK) at 1:500 dilution in phosphate buffer saline (PBS) used to detect M2 macrophages and monoclonal anti-FoxP3 antibody (clone 236A/E7, Abcam, Cambridge, UK) at 1:100 dilution in PBS used to detect Treg. Visualization was performed by using diamine-benzidine (DAB) (Abcam, Cambridge, UK) at 1:50 dilution in DAB substrate according to the manufacturer’s instructions. Liver mouse tissue was used for M2 macrophage positive control and human tonsil for Treg positive control.

*Immunohistochemistry (IHC) assessment*

For CD163 in M2 macrophages, each section was screened at low-power magnification to identify the areas with the highest staining density. The positive expression of CD163 was defined by a granular cytoplasm or a cytoplasmic and membrane staining pattern. The expression CD163 was classified into four grades (0-3): negative (0), no staining; scant (1), a small amount of scattered staining; focal (2), concentrated staining with an irregular and non-continuous focus; and abundant and (3), concentrated stains with an extensive and continuous focus. This classification system was similar to the system used in a previous study (Fujii et al., 2012) as seen in Figure 2. Based on the result of the expression levels of CD163, they were classified into the “high” group for scores of 2–3 and the “low” group for scores of 0–1 (Yu et al., 2018). The expression of Foxp3 showed the staining in the nucleus of T cells. Five high-power fields (HPF) were randomly selected. The number of positive cells were counted in each HPF, and the expression data identified as the mean of the number of positive cells in 5 HPF (Wang et al., 2017). Each section was scored independently by two pathologists (Ardiyan et al., 2020). The average values from the two pathologists were used for further analysis.

*Statistical analysis*

The correlation between M2 macrophage and Treg were analyzed using Spearman correlation. The analysis of correlation between the M2 macrophage and Treg with TNM and clinical stage were analyzed using Chi square tests. All tests were performed using SPSS 18 (IBM Corp., Armonk, NY). All p-values presented were two-sided with p≤ 0.1 defined as statistically significant.

## Results

*Demographic and clinicopathological features of NPC patients*

Fifty (50) patients with relatively complete clinicopathological data extracted from medical records were included in this study. Seventy-four percent (74%; 35/50) were male patients and 26% (13/50) were female patients with a median age of 51 years (range, 18-79 years). Forty-six percent (46%; 23/50) of the patients had T1- T2 tumor size, and 54% (27/50) had T3-T4 tumor size. Ten percent (10%; 5/50) had negative node involvement (N0) and 90% (45/50) were N1-3. Ninety-six percent (96%; 48/50) of the patients suffered from locoregional recurrence and 4% (2/50) suffered from distant metastasis. Based on the staging classification, 8% (4/50) of the patients had stage II disease, and 92% (46/50) had stage III-IV disease (Table 1).

*Immunohistochemistry (IHC) Analysis of M2 Macrophage and Treg*

**Figure 1:**
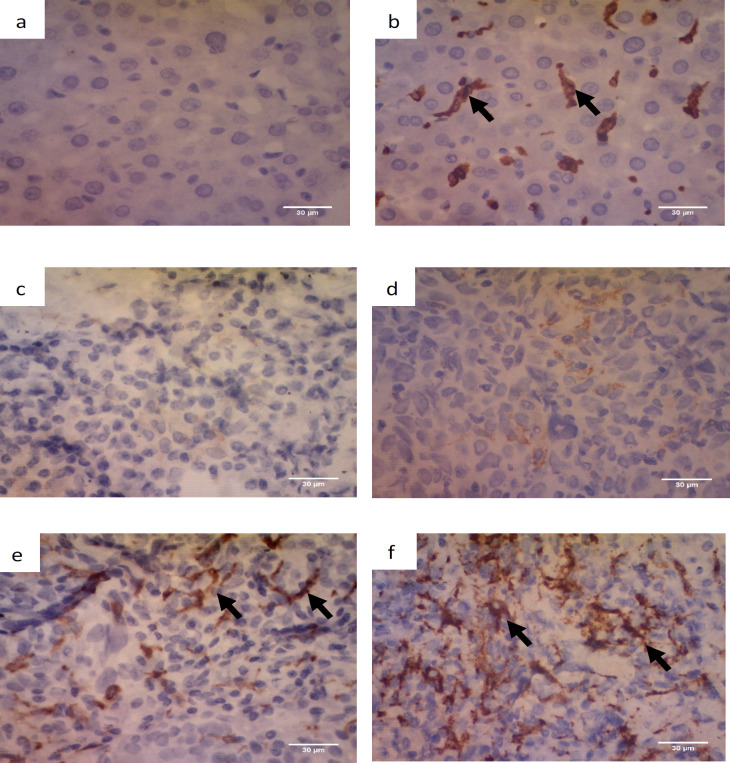
Immunohistochemistry of M2 Macrophage Using Anti-CD163 Antibody. Positive staining was shown on the membrane and cytoplasm (black arrow). The images were at 400x magnification. a. negative control (liver mouse) without anti-CD163 antibody, b. positive control (liver mouse) with anti-CD163 antibody, c. negative (0 score), no staining; d. scant (1 score), a small amount of scattered staining; e. focal (2 score), concentrated stains with an irregular and non-continuous focus; and f. abundant (3 score). Scale bar, 30 µm

**Figure 2:**
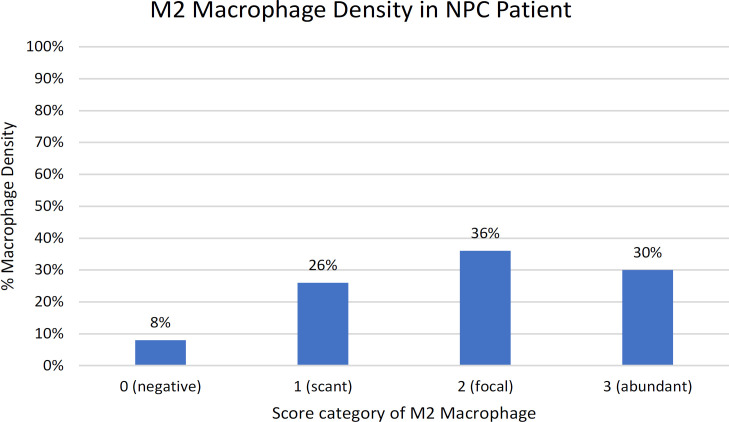
M2 Macrophage (CD163) Density in Patients with NPC. Eight percent (8%; 4/50) of patients were negative result of CD163, 26% (13/50) were scant grade, 36% (18/50) were focal grade and 30% (15/50) were abundant grade

Immunohistochemistry detection of M2 macrophage (CD163 expression) in the NPC tissue samples can be seen in Figure 1. Eight percent (8%; 4/50) of the samples had negative result, 26% (13/50) had scant grade, 36% (18/50) showed focal grade, and 30% (15/50) had abundant grade (Figure 2). According to the positivity score of CD163, 34% (17/50) of the samples were classified into low group, while 66% (33/50) were classified into the high group. 

**Figure 3:**
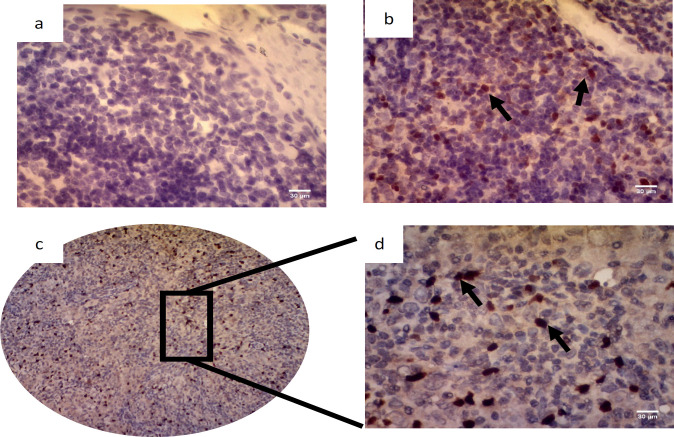
Immunohistochemistry of Treg Using Anti-FoxP3 Antibody. Positive staining was shown in the nucleus (black arrow). The images were taken with a 400x magnification. a. negative control (human tonsil) without anti-FoxP3 antibody, b. positive control (human tonsil) with anti-FoxP3 antibody, c. positive result in NPC tissue (100x magnification), and d. positive result in NPC tissue (400x magnification). Scale bar, 30 µm

**Figure 4:**
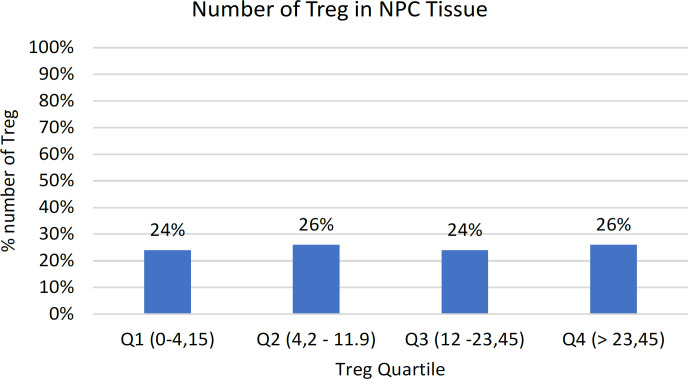
Quartile Group of Tregs. The results were grouped into four categories: Quartile 1 (0 – 4.15), Quartile 2 (4.2-11.9), Quartile 3 (12-23.45), and Quartile 4 (> 23.45). Twenty-four percent (24%; 12/50) of patients were in Q1, 26% (13/50) were in Q2, 24% (12/50) were in Q3 and 26% (12/50) were in Q4

Detection of Treg (FoxP3 expression) in the NPC tissue samples is shown in Figure 3. The average number of Tregs in NPC tissue was 16.31 ±16.07. The median value obtained was 12.3; with the minimum value was 0 and the maximum was 74. The results were grouped into four categories: Quartile 1 (0-4.15), Quartile 2 (4.15-11.9), Quartile 3 (12-23.45), and Quartile 4 (> 23.45). Twenty-four percent (24%; 12/50) of the patients were in Q1, 26% (13/50) were in Q2, 24% (12/50) were in Q3 and 26% (13/50) were in Q4 (Figure 4). The Cronbach Alpha values of the IHC interpretation of M2 macrophage and Treg tests were done to assess the reliability between two observers.

*Correlation between M2 macrophage and Treg; and correlation between M2 macrophage, Treg with TNM and Clinical stages*

Spearman’s correlation revealed a positive correlation between M2 macrophages and Treg in NPC tissue (r=0.469, p= 0.001). The distributions of the characteristics of the sample sets within the scoring groups are summarized in Table 2. The presence of M2 macrophages and Treg was significantly correlated with T (tumor size) (p= 0.091 for M2 and p=0.022 for Treg). The presence of M2 macrophages and Treg was also significantly correlated with clinical stage (p= 0.030 for M2 and p= 0.002 for Treg) (Table 2). 

**Table 1:** Demographic and Clinicopathologic Data of NPC Patients

Demographic	Patient (n=50)	Patient (%)
Gender (n)		
Male	37	74
Female	13	26
Age (yr)		
18-40	11	22
41-60	30	60
>60	9	18
T status		
T1-T2	23	46
T3-T4	27	54
N status		
N0	5	10
N1-N2-N3	45	90
M status		
M0	48	96
M1	2	4
Stage		
II	4	8
III	23	46
IV	23	46

**Table 2:** The Correlation among the Clinical Parameters, M2 Macrophage and Tregs (n=50)

Clinical Parameters	M2 Macrophage			Treg		
	Low (n=17)	High (n=33)	p-value	≤ median (n=24)	> median (n=26)	p-value
T stage						
T1-T2	5	18	0.091*	7	16	0.022*
T3-T4	12	15		17	10	
N stage						
N0	2	3	0.765	1	4	0.187
N1-N3	15	30		23	22	
M stage						
M0	17	31	0.3	22	24	0.149
M1	0	2		2	0	
Clinical Stage						
II	0	4	0.030*	0	4	0.002*
III	5	18		7	16	
IV	12	11		17	6	

## Discussion

Nasopharyngeal carcinoma (NPC) is a common cancer of the human head and neck which is associated with genetic predisposition, Epstein-Barr virus (EBV) infection, and environmental factors. Global cancer statistic 2018 report found 129,079 cases of nasopharyngeal cancer worldwide, and 93,416 (72.37%) were in males and 35,663 (27.63%) were in females with a ratio of 2.62:1. In Southeast Asia, from a a total of 34,681 new cases, there were 25,895 cases (74.67%) diagnosed in males and 8,786 cases (25.33%) in females, with a ratio of 2.95:1 (Bray et al., 2018; Duc and Ai, 2020). Data in the Indonesian Cancer Registry reported that NPC is more common in males, and is the second most common type of cancer in men after lung cancer. From the 50 patients included in this study, 74% (37/50) patients were male and 26% (13/50) were female. Twenty-two percent (22%; 11/50) of the patients were aged between 18-40 years, 60% (30/50) were 41-60 years old and 18% (9/50) were over 60 years old. The number of patients increased after the age of 30 years, and reached a peak between 50 and 59 years. Our study results were similar to studies in high-incidence areas in China (Parkin et al., 2005; Bray et al , 2018). 

The most common NPC subtype in endemic areas including Indonesia is the undifferentiated carcinoma subtype, which is 100% related to EBV infection. The involvement of EBV infection in NPC cells causes an interaction between EBV and host cells. The interaction between EBV RNAs (EBERs) and TLR3 on host cells leads to the increase of inflammatory cytokine expression, including tumor necrosis factor-alpha (TNFα), IL-6 and IL-1α. Those cytokines potentially recruit and activate macrophages, leading to a pro-tumorigenic microenvironment for solid tumor growth (Li et al., 2015). 

Macrophages in the tumor microenvironment serve as tumor facilitators for cell migration and invasion, extracellular matrix degradation, and angiogenesis. Macrophages may be differentiated into two types: type 1 (M1 macrophage), which is activated classically and the alternative type 2 (M2 macrophages) (Mantovani et al., 2002). M1 macrophage is considered as proinflammatory and antitumoral, while M2 macrophages encourage tumor development, possessing immunosuppressive and protumoral effects (Galdiero et al., 2013; Ruffellet al., 2012). M2 macrophage can recruit mature Treg and modulate the conversion of naive T cells to Treg (Mantovani et al., 2002). Tregs play a role in immunosuppression and immune escape in tumor microenvironment (Chen et al., 2019). Research on M2 macrophage and Treg in EBV related NPC has not yet been described. Several studies reported that M2 macrophage and Treg are closely related with worse survival and poor prognosis in several types of cancer. M2 macrophage and Treg can be important indicator factors to determine therapeutic targets (Ooft et al., 2018; Wang et al., 2017). 

The results of this study indicated that M2 macrophage and Treg have a positive correlation while the amounts of 46.9%, and 53.1% of the correlation were affected by other factors, possibly by the EBV oncogene Latent membrane Protein 2 (LMP1) and Epstein-Barr Nuclear Antigen (EBNA1). Wang et al., (2017) reported that NPC cell line culture (CNE1, CNE2, and 5-8F) can recruit M2 macrophage and furthermore, Treg was recruited by M2 macrophage. Their study also showed that M2 macrophage have a positive association with Treg cells. Macrophage secretes chemokines such as CCL22, CCL17, and CCL18 to recruit Treg and modulate the immune response during the tumorigenic process (Curiel et al., 2004; Mantovani, 2008). Another factor that can recruit Treg in tumor area is LMP1. LMP1 can recruit Tregs through the activation of NF-kB signaling that will lead the upregulation of the CCL20 chemokine (Baumforth et al., 2008). High CCL20 expression increases the migration of Tregs cells towards the tumor (Baumforth et al., 2008; Tsao et al., 2014). Furthermore, LMP1 can also induce Tregs to secret IL-10 (Marshall et al., 2003) that plays a role in immune suppression. Besides M2 macrophage and LMP1, Wang et al., (2020) also report that EBNA1 induces the accumulation of Tregs through the conversion of naïve T cell into Tregs via upregulated TGF-β1 and CCL20 which will induce EBV-directed migration, and polarized-M2 macrophage to convert naïve T cells into Tregs. 

Previous study showed that CD163 is a highly specific marker of M2 macrophages and has been studied in several aggressive tumors. The increased expression of CD163 was significantly associated with a poor prognosis in various cancers (Clear et al., 2010; He et al., 2014; Kridel et al., 2015; Tiainen et al., 2015). Yu et al., (2018) also reported that higher expression of CD163 predicted worse survival of NPC patients. This is probably due to M2 macrophages promoting tumor growth. Tumor associated macrophages (TAMs) that are dominated by M2 are known to induce Tregs recruitment and differentiation in the tumor microenvironment (Daurkin et al., 2011; Denning et al., 2007). M2 macrophage and Treg form a special niche, which promotes tumor progression (Kitamura et al., 2015). In our study, M2 macrophage and Treg were significant correlated with tumor size (T status) and clinical stages, but no correlation was found with the involvement of the lymphatic nodes nor with distant metastasis of NPC. 

This study had several limitations, including the small number of the samples which may influence the results. This study only focused on the presence of M2 and Treg and their association with severity. Several parameters, including other cells in NPC tumor microenvironment (inflammatory cells, cancer associated fibroblasts, and endothelial cells), and EBV or cellular proteins that play a role in NPC growth were not included in this study. Since most of the samples (92%) were at the advanced stage NPC (III and IV), it is difficult to determine the correlation between the presence of M2 macrophage and Treg with TNM status and clinical stage objectively. 

In conclusion, the presence of M2 macrophage significantly correlates with the presence of Treg in EBV-related NPC tumor microenvironment. M2 macrophage and Treg were significantly correlated with tumor size and clinical stage. For further study, it is necessary to look at other parameters in the tumor microenvironment that play a role in tumor progression, and also assess the survival rate and other clinicopathology features as prognostic factors.

## Author Contribution Statement

SHA, YNA and DKP were involved in planning. SHA, IM, NSNH drafted the manuscript and designed the figures. CH collected samples from the patients with NPC. EKD analyzed the IHC results. SHA and JF performed the statistical analysis. SHA, WA and DKP wrote the discussion and conclusions. After the first draft, all of the author discussed and commented on the manuscript. All authors read and approved the final version of the manuscript. SHA and DKP finalized the manuscript to be ready for submission.

## Funding statement

This work was supported by the grant from Society Funding (Dana Masyarakat/DAMAS) Faculty of Medicine, Public Health and Nursing Universitas Gadjah Mada 2019 and Doctorate Dissertation Research (Penelitian Disertasi Doktor/PDD) 2020 No. 2071/ UN1/DITLIT/DIT-LIT/PT/2020, Ministry of Education & Culture Republic of Indonesia.

## Data statement

Some data of this study were used for completing the thesis of Yustina Nuke Ardiyan and will be used in the doctoral study of Siti Hamidatul ‘Aliyah.

## Conflict of interest

The authors declare no potential conflict of interest.
